# Immediate weight-bearing after tibial plateau fractures internal fixation results in better clinical outcomes with similar radiological outcomes: a randomized clinical trial

**DOI:** 10.1007/s00264-025-06443-1

**Published:** 2025-02-18

**Authors:** Mariam Abdel-azim Ibrahim, Mohamed M. A. Moustafa, Jean-Michel Brismée, Osama Farouk, Mohammad Kamal Abdelnasser, Hatem Galal Said, Troy L. Hooper, Mohamed Abdelmegeed, Ayman F. Abdelkawi

**Affiliations:** 1 Orthopedic Rehabilitation Unit, Department of Orthopedic and Trauma Surgery, Assiut University Trauma Hospital, Assiut University, Assiut, Egypt; 2 Department of Orthopedic and Trauma Surgery, Assuit University Trauma Hospital, Faculty of Medicine, Assiut, Assiut, Egypt; 3 Center for Rehabilitation Research, School of Health Professions, Texas Tech University Health Sciences Center, Lubbock, Texas, Lubbock, USA; 4 Department of Orthopedic Physical Therapy, Faculty of Physical Therapy, Cairo University, Cairo, Cairo, Egypt; 5 Department of Allied Health and Kinesiology, School of Health Sciences, Hofstra University, New York, New York, USA; 6 Clinic for Orthopedics, Trauma, Shoulder Surgeries and EndoprostheticsRHÖN-KLINIKUM AG, Bad Neustadt, Germany

**Keywords:** Physical therapy, Intra-articular knee fractures, CT-Scan, Accelerated rehabilitation, Rasmussen score

## Abstract

**Purpose:**

To investigate the effects of adding immediate weight-bearing to tolerance into a post-operative rehabilitation program for surgically treated Tibial Plateau (TP) fractures on clinical and radiological outcomes.

**Methods:**

A randomized control trial. 106 Patients were recruited following open reduction internal fixation (ORIF) TP fracture, with 54 patients meeting the criteria for inclusion. Patients were assigned randomly into one of two groups: (1) the traditional group (TG) and (2) the weight-bearing group (WG). The TG was given the non-weight-bearing (NWB) rehabilitation protocol for six weeks. The WG was allowed immediate weight-bearing, and the same therapeutic exercise program was given to both groups. The dependent variables, including clinical and radiological measurements, were recorded six weeks, three months, and six months after the surgery.

**Results:**

A total of 45 patients (11 women and 34 men), with a mean age of 43 *±* 14 years, completed the study. There were significant differences between groups in favor of the WG at 6-months for the total clinical Rasmussen score (*p* =.002) as well as for the pain (*p* =.005), walking capacity (*p* =.002), and knee ROM (*p* =.047). We found neither difference between groups regarding radiological CT- Scan and X-ray measures nor Rasmussen’s radiological scores (*p* =.854). Fracture type (Schatzker I-IV) did not affect any radiological measures between the groups. Four of 45 patients had intra-articular collapse, three in TG and one in WG (*p* =.571).

**Conclusion:**

Immediate weight-bearing as tolerated after ORIF of TP fractures (Schatzker I-IV) resulted in better clinical outcomes with no significant differences in the radiological measures.

**Supplementary Information:**

The online version contains supplementary material available at 10.1007/s00264-025-06443-1.

## Introduction

Tibial plateau (TP) fractures account for approximately 1% of all fractures in adults and can result in disability or decreased overall function [1]. Understanding the nature of the injury and applying the appropriate fixation to achieve ideal reduction and stability is critical [2].

For optimal recovery, the post-operative protocol should set weight-bearing status near the upper limit to support a swift return to pre-injury condition. However, it must also be safe enough to avoid complications regarding reduction in quality and joint overloading. There is some growing evidence that surgeons may safely allow early or immediate post-operative weight-bearing by using locking plates for the surgical management of TP fractures [3, 4]. To our knowledge, no prospective clinical study has investigated the effect of immediate weight-bearing compared to a non-weight-bearing (NWB) rehabilitation protocol following TP fracture surgical fixation. Therefore, the study aimed to investigate the difference in clinical and radiological outcomes following surgical fixation of TP fracture types I, II, III, and IV Schatzker classification [5].

## Methods

### Research design(s)

A randomized control trial. The trial was registered at https://clinicaltrials.govNCTxxxxNCT05502679. The study was carried out according to the Helsinki Declarations, and written informed consent was obtained from all participants. The CONSORT recommendations for reporting an RCT trial were followed (supplementary file 1). Faculty of Medicine, Assiut University IRB approved this study protocol, no17200756.

### Reliability of measurements testing

Radiological measurement intra-rater reliability was tested for one orthopedic surgeon with more than 15 years of experience. The intraclass correlation coefficient ICC estimates and their 95% confidence intervals were calculated based on a mean-rating (k = 4), absolute-agreement, two way mixed-effects model. (ICC) Model 3,4 was 0.99 (CI = 0.996 − 0.999).

### Patients and investigators

A total of 106 Patients were recruited following open TP fracture and internal fixation performed at Assiut University Trauma Hospital– Level One Trauma Center, Egypt, from June 2022 until January 2024. Inclusion criteria were: women and men (18 to 65 years of age) with the diagnosis of traumatic TP closed fracture, treated by ORIF, Schatzker classification type I to IV, operated on by orthopaedic surgeons with at least five years of experience in surgeries using pre-contoured and standard locking compression plates [6]. Exclusion criteria were any fractures Schatzker type V and VI, open fractures, ipsilateral or contralateral upper or lower limb concomitant injuries that prevented either weight-bearing or using crutches, percutaneous fixation or with external fixation, delayed surgery for more than seven days after the injury, post-operative complications that prevent second day discharge or interfere with patient ability to ambulate immediately, non-ambulatory Patients before the injury.

### Testing sequence

Each patient received an explanation of the study, and written informed consent was obtained. A randomization plan generator by an investigator not involved in the data collection was used to assign each patient to one of two interventions: (1) Immediate weight-bearing Group (WG) *n* = 19 or (2) Traditional Group (TG) *n* = 26. Post-operative weight-bearing was prescribed, either immediate and unrestricted weight-bearing as tolerated (WG) or six weeks of NWB (TG). Patients were mobilized from the first post-operative day or when medically fit. Patients in WG proceeded from walkers to crutches or walking sticks, according to their ability, under the physiotherapists’ supervision. Patients in the TG were taught to walk three point gait with a standard walker and two axillary or elbow crutches and did not progress beyond their prescribed restriction for six weeks.

A research coordinator not involved in data collection was responsible for patient enrollment, group assignment, and data entry. All Patients were treated in the hospital on the first day following the surgery (baseline) and were given the respective rehabilitation protocol, which included daily home-based exercise prescription.

**Fig. 1:**
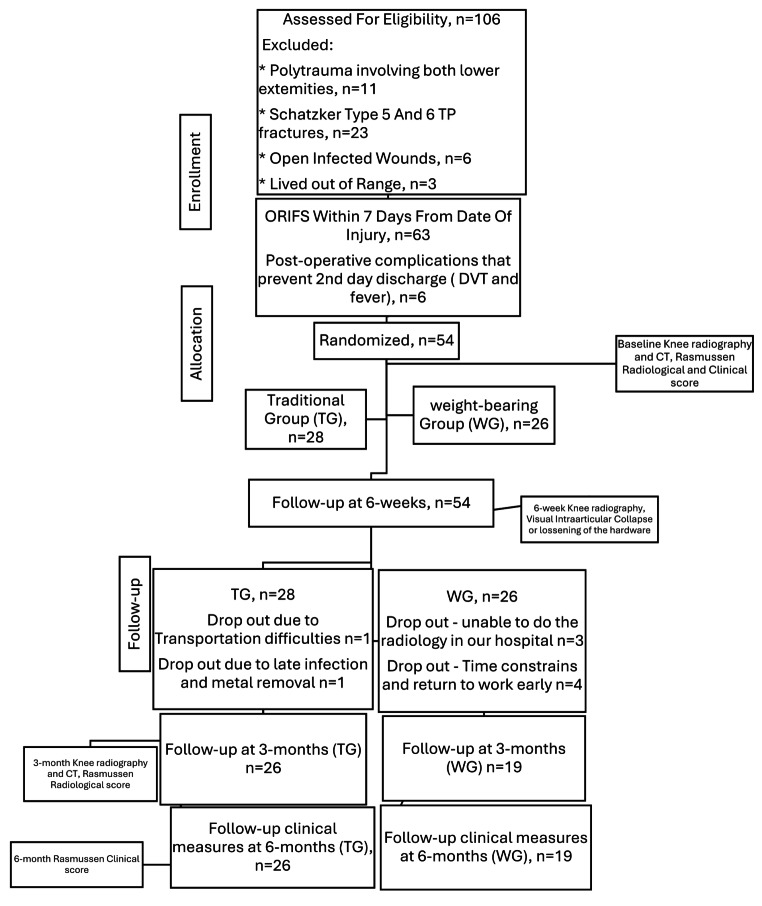
Flow diagram of the study. ORIF: Open reduction internal fixations; DVT: Deep Vein Thrombosis

Two orthopedic surgeons with a mean of 15 years of experience in trauma surgery who classified the fractures and three physiotherapist investigators who assessed the clinical dependent variables were blinded to the group assignment. All patients underwent an affected knee supine Anterior-Posterior (AP) X-ray and CT-Scan post-operatively before being discharged home. At six weeks after the surgery, Patients in TG were asked to start weight-bearing on the affected lower extremity, and both groups underwent another x-ray of the affected knee to detect any visual intraarticular collapse or hardware loosening. At 3-months, both groups underwent affected knee AP X-ray and CT for final radiology follow-up (Fig. 1).

### Outcomes measures

#### Imaging studies

One orthopaedic surgeon blinded to the patients’ group assignments measured all radiological parameters for both groups at baseline and three month follow-up.

*Knee X-ray measurements*.

1. The medial proximal tibial angle (MPTA) was measured as the inclination between the axis of the TP articular surface and the proximal tibial anatomical axis in the coronal plane [7].
2. Visual intra-articular collapse or loosening of the hardware was collected during the six week follow-up. Articular collapse is defined as decreased radiologic joint space and increased articular damage risk [8, 9].

*Knee CT-Scan measurements*.

The coronal cut used for the measures was the widest, and the plane of sagittal reconstruction was perpendicular to the posterior distal femoral intercondylar axis. The tibial axis was identified on the midsagittal cut at the centre of the medullary canal. The investigator ensured measurement consistency by using the same cuts for each patient at baseline and during the final follow-up.

**Fig. 2:**
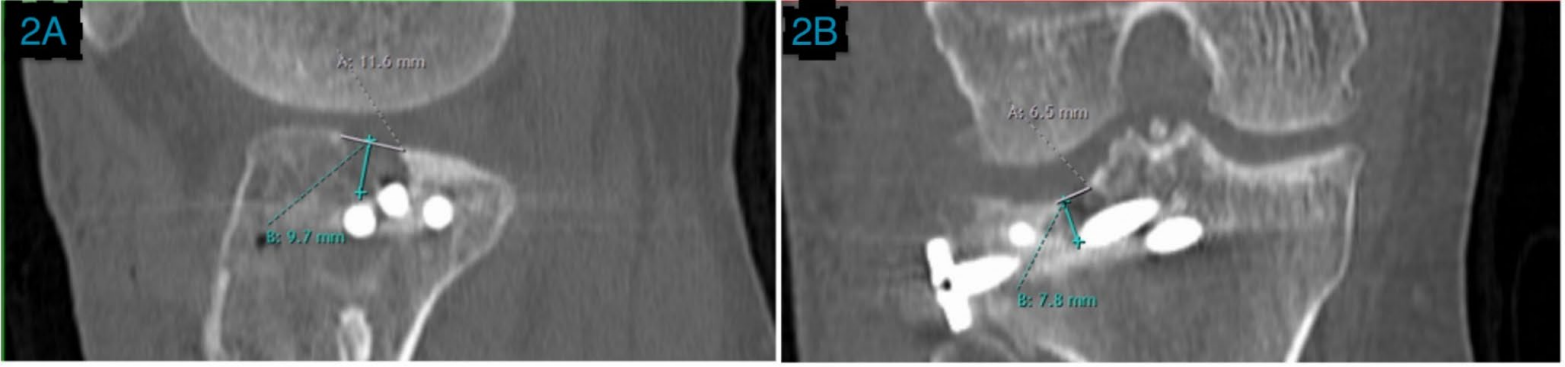
Horizontal fracture gap and depression **2A)** Coronal plane: The gray line represents the horizontal fracture gap, and the turquoise line represents the articular depression. **2B)** Sagittal plane: The gray line represents the horizontal fracture gap, and the turquoise line represents the articular depression

**Fig. 3:**
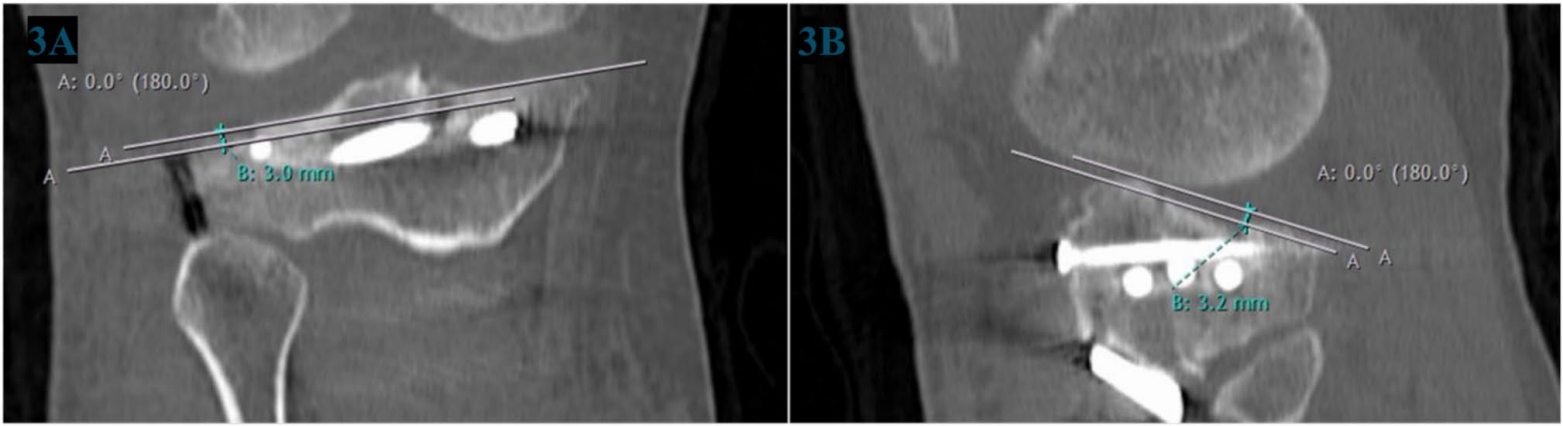
Joint Step off. The gray lines are parallel to the slope in the sagittal plane. **3A)** Coronal plane: Joint Step off. **3B)** Sagittal plane: Joint Step off

**Fig. 4:**
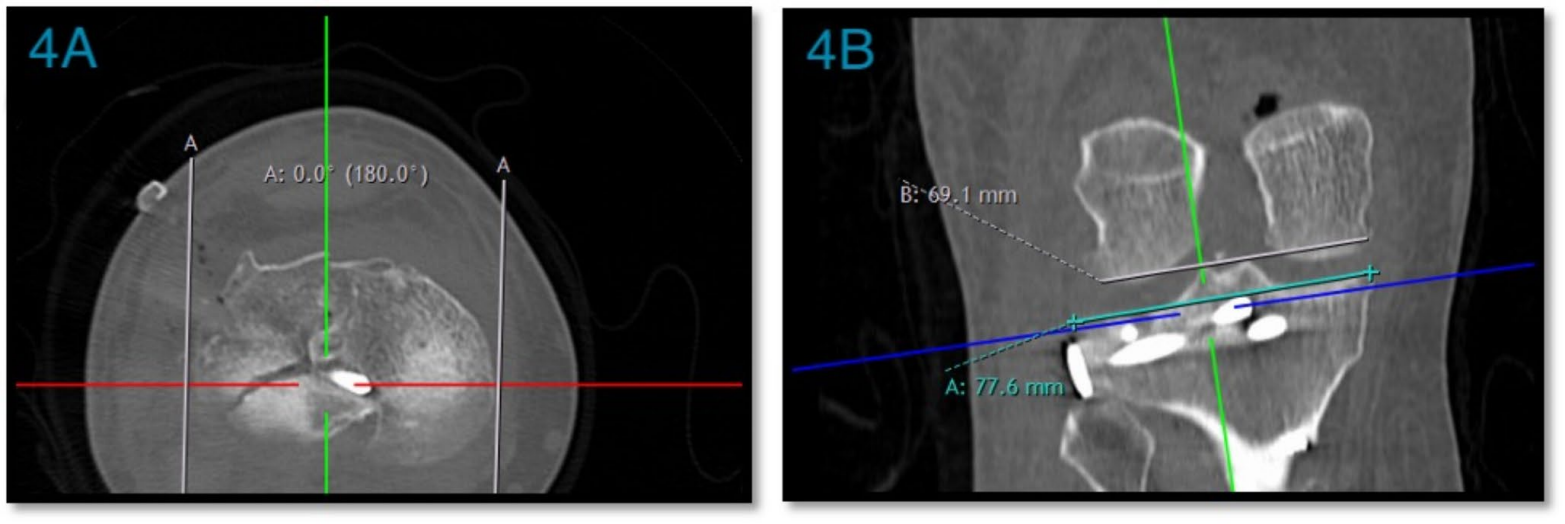
Tibial Plateau Width and Condylar Widening. The dark blue line is positioned at the highest point of the proximal tibia **4A)** The red line and **4B)** the turquoise line represent the broadest part of the tibial plateau. At the same level, the gray line indicates the femoral condylar width, with condylar widening defined as the difference between the tibial plateau and femoral width

**Fig. 5:**
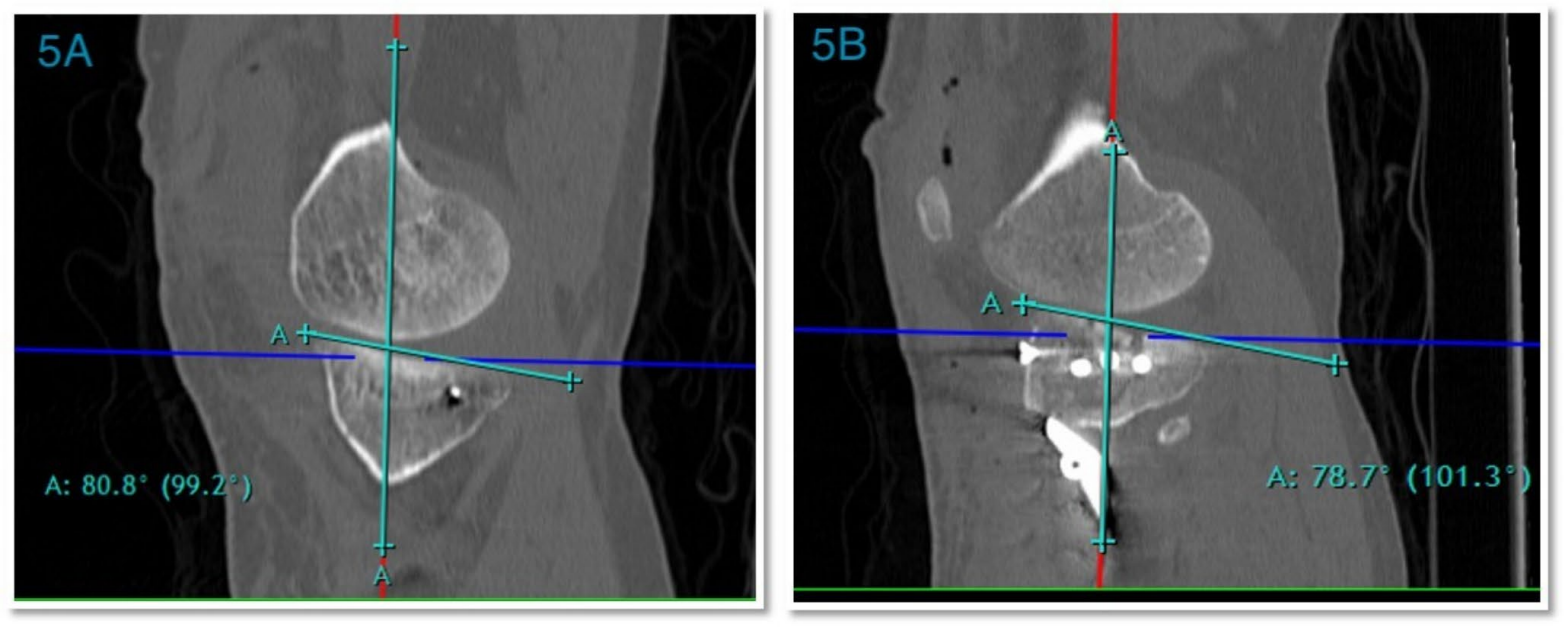
Tibial Slope, The red line adjusted on the center of the medullary canal and the highest point of the lateral TP measurement was done at the middle of the TP opposite the lowermost end of the femoral condyle **5A)** Medial tibial slope **5B)** Lateral tibial slope

1. Articular Depression (coronal or sagittal): measured as the vertical distance from the displaced fracture fragment to a line drawn through the same affected articular plateau (Fig. 2).
2. Horizontal fracture gap (coronal or sagittal): The horizontal distance between fracture fragments (Fig. 2).
3. Joint Step-off (coronal and sagittal): measured as the vertical distance from the displaced fractured articular surface to a line drawn through the unaffected articular plateau [10] (Fig. 3).
4. TP width: the maximum TP width at the widest cut [11] (Fig. 4).
5. Condylar widening measurement: measured as the distance between these two lines perpendicular to the medial tibial articular surface, one along the most lateral aspect of the distal femoral condyle and the other along the most lateral aspect of the proximal tibia, and it is equal to the difference between tibial condylar width and femoral condylar width [10] (Fig. 4).

6) The Tibial Slope is measured in a sagittal plane. It is defined as the angle between the line perpendicular to the tibial shaft axis and the posterior inclination of the TP. During a dynamic manoeuvre, it affects the amount of anterior tibial translation and anterior cruciate ligament (ACL) strain. The slope of each articular surface is measured concerning the lines transferred from the midsagittal sections relative to the mechanical tibial axis [12].
  - The lateral plateau slope is measured in the center of the lateral plateau parallel to the uppermost portion of the articular surface. The average angle is (81.5–87.9) (Fig. 5a).
  - The medial plateau is more concave from anterior to posterior; the slope measurement is identified by drawing a tangent to the uppermost superior-anterior and posterior cortical edges. The average angle is (80.1–87.5) (Fig. 5b).

#### Rasmussen measurements

The Rasmussen score is a system used to evaluate the outcome of surgical intervention for intra-articular fractures. This score assesses both radiological and clinical outcomes.

*Radiological Rasmussen score*: The radiological section evaluates reduction quality and alignment through the degree of joint depression, condylar widening, and angulation. It is scored out of 18 points. A higher score indicates a better radiological outcome, indicating that the fracture was well-reduced and aligned with minimal residual deformity.

*Clinical Rasmussen score*: The clinical portion assesses the patient’s functional outcome post-surgery. It also typically ranges up to 30 points based on pain level, walking capacity, range of motion (knee flexion/extension), and knee joint stability. The interpretation is that a higher score reflects less pain, better mobility, and overall joint stability [13]. The final Rasmussen clinical scores were graded at six month intervals by two physical therapists blinded to the groups the patients were assigned to.

### Data analysis

Jamovi Version 2.5 Computer Software was used for statistical analysis. The level of significance was presumed at *p* <.5. Patient measurements were described as the mean ± standard deviation, the minimum and maximum values for continuous data, and frequency counts for qualitative data. The mean differences between the baseline and post-intervention measurements (6-week, 3- and 6-month) and the differences in their changes over time between the (WG) and (TG) groups with the 95% confidence intervals were measured. Nonparametric statistics were used because most data did not meet normality assumptions, and for between-groups (TG versus WG) comparisons, the groups were of unequal sizes. Therefore, we used the Wilcoxon Signed Ranks test for within-group comparisons, Chi-Square, and Kruskal Wallis analysis of variance (ANOVA) to assess differences. A prior power analysis was performed using an online calculator. 52 patients were estimated to detect a 5% significance level with an 80% confidence interval, a significant difference between the TG and WG for clinical outcomes in patients diagnosed with TP fractures surgical fixation. We recruited 106 patients to account for attrition.

## Results

A total of 45 patients: men = 34(75.6%) with a mean age of 43 *±* 13.9 years (range 18–65) and a mean baseline Rasmussen radiological score of 14.4 *±* 2.93 (range 6–18) and clinical Rasmussen score of 9 ± 2.22 were included in our study statistical analysis after nine patients dropped-out during the three and six month follow-up periods due to time constraints, transportation difficulties and metal removal surgery but not due to any postoperative late complications. The demographics of the 45 patients who completed the study are presented in (Table 1). 35 patients suffered a fracture of the lateral tibial condyle and ten of the medial tibial condyles in traffic accidents. Fractures were classified according to the Schatzker classification. The fractures observed in this study were classified as Schatzker I (*n* = 12; 27.0%), Schatzker II (*n* = 11; 25.0%), Schatzker III (*n* = 11; 25.0%), and Schatzker IV (*n* = 10; 23.0%).

**Table 1:** Baseline characteristics of the study patients by group

Variables	WG Group	TG Group	*p* values
Age (years)	42.3 *±* 12.9	43.3 *±* 14.9	0.820
Gender	*N* = 19, 14♂ & 5♀	*N* = 26, 20♂ & 6♀	0.803
Fracture Type	Type 1, *n* = 5 (11.4%)	Type 1, *n* = 7 (15.9%)	0.590
	Type 2, *n* = 6 (13.6%)	Type 2, *n* = 5 (11.4%)	
	Type 3, *n* = 3 (6.8%)	Type 3, *n* = 8 (18.2%)	
	Type 4, *n* = 5 (11.4%)	Type 4, *n* = 5 (11.4%)	
Baseline Rasmussen radiological scoreξ	13.9 *±* 3.68	14.7 *±* 2.23	0.766
Baseline Rasmussen clinical score ξ	8.89 *±* 2.64	9.46 *±* 1.88	0.149

WG = WB Group; TG = Traditional Group; ξ indicates Mann-Whitney U test; Mean ± Standard Deviation; ♂ = males; ♀ = females; * indicates statistical significance

### Radiological measurement outcomes

#### Between droups analysis

We found no statistical difference between groups regarding radiological CT-scan and X-ray measures. There was also no statistical difference in any radiological measures between the groups for the effect of fracture type. Four out of 42 patients had intra-articular collapse, three in TG and 1 in WG (*p* =.571). Five patients (2 in TG and 3 in WG) demonstrated excellent initial alignment, 33 (21 in TG and 12 in WG) demonstrated good initial alignment, six (2 in TG and 4 in the WG) demonstrated fair initial alignment, with either sagittal or coronal alignment greater than 5 degrees outside the accepted range and no patient demonstrated poor initial alignment score. No patient went from excellent or good initial alignment to poor final alignment. No patient required unplanned secondary surgical procedures. One patient underwent metal removal after the union. Patients demonstrated neither implant bending nor failure nor required return to the operating room for alignment loss or revision internal fixation.

#### Within groups analysis

There was no difference within groups for all radiological outcomes, except for the horizontal gap in the frontal and sagittal planes (*p* =.002) for the TG (Table 2).

**Table 2:** Within-group difference from baseline to the final follow-up

Variables	TG			*P* value	WG			*P* value
	Initial	3-month	Differences (3-month– initial)		Initial	3-month	Differences (3-month– initial)	
Proximal Medial Tibial Angle(º)	86.69 *±* 3.58	86.15 *±* 3.60	− 0.54	0.664	87.04 *±* 1.34	87.63 *±* 1.82	0.59	0.138
Depression In Sagittal Plane (mm)	3.01 *±* 2.44	3.20 *±* 2.44	0.19	0.659	3.10 *±* 2.17	3.73 *±* 3.14	0.63	0.099
Depression In Frontal Plane (mm)	3.48 *±* 2.52	3.48 *±* 2.59	0	0.659	2.81 *±* 2.55	3.47 *±* 3.19	0.66	0.255
Horizontal Gap in Frontal Plane (mm)	4.74 *±* 5.29	2.90 *±* 3.65	-1.84	0.002*	4.33 *±* 4.05	4.20 *±* 5.65	− 0.13	0.225
Horizontal Gap in Sagittal Plane (mm)	5.49 *±* 5.85	5.42 *±* 8.95	− 0.07	0.002*	4.06 *±* 3.52	4.18 *±* 5.63	0.12	0.092
Joint Step Off in Frontal Plane (mm)	2.03 *±* 2.13	2.42 *±* 2.51	0.39	0.066	2.57 *±* 2.44	1.99 *±* 2.2.34	0	0.794
Joint Step Off in Sagittal Plane (mm)	1.83 *±* 2.28	2.38 *±* 2.85	0.55	0.066	1.98 *±* 1.85	1.98 *±* 1.93	0	0.104
Tibial Plateau Width (mm)	76.07 *±* 5.98	76.08 *±* 5.30	0	0.054	74.53 *±* 5.95	75.33 *±* 6.54	0.80	1.00
Condylar Widening(mm)	6.81 *±* 3.16	7.12 *±* 3.28	0.31	0.561	7.72 *±* 3.45	7.63 *±* 3.70	0.09	0.658
Lateral Tibial Slope (º)	84.17 *±* 5.06	85.00 *±* 5.02	0.83	0.274	81.89 *±* 5.89	81.49 *±* 6.02	0.4	0.088
Medial Tibial Slope(º)	84.25 *±* 6.00	83.62 *±* 5.09	− 0.63	0.327	81.79 *±* 3.28	81.84 *±* 5.84	0.05	0.408
Rasmussen Radiological Score	14.72 *±* 2.22	14.32 *±* 2.28	0.40	0.090	13.89 *±* 3.68	13.89 *±* 3.91	0	0.990

WG = WB Group; TG = Traditional Group; Mean ± Standard Deviation; (mm) = Millimeter; (º) = angle degrees; Repeated measure ANOVA; *indicates statistical significance

### Clinical Rasmussen score

There were significant differences between groups in favor of the WG at six months for the total clinical Rasmussen score (*p* =.002), pain (*p* =.005), walking capacity (*p* =.002), and functional ROM (*p* =.047). 73.7% of the WG patients scored “excellent” in Rasmussen clinical score at six months compared to 34.6% for TG (Table 3).

**Table 3:** Rasmussen clinical total scores at 6-month follow-up

Group	TG	WG	*P* value	Effect sizes	η² interpretation
Pain	3.58 ± 2.24	5.16 ± 1.39	0.005*	0.1821	Large effect
Walking Capacity	3.96 ± 1.54	5.32 ± 1.34	0.002*	0.2221	Large effect
Knee Extension	5.15 ± 1.42	5.79 ± 0.918	0.046*	0.0904	Medium effect
Available ROM	4.23 ± 1.30	5.00 ± 0.745	0.047*	0.0901	Medium effect
Knee Stability	4.77 ± 1.39	5.53 ± 0.964	0.027*	0.1106	Large effect
Rasmussen Clinical Score	21.69 ± 6.89	26.79 ± 4.66	0.002*	0.2093	Large effect

TG = Traditional Group; WG = WB Group; Mean ± Standard Deviation; η²= Effect size; * indicates statistical significance

### Radiological Rasmussen score

There were no significant differences between groups at 3-months for the total radiological Rasmussen score (*p* =.854) or the condylar widening (*p* =.871).

## Discussion

Our findings support immediate weight-bearing as there were no between-groups differences in radiological outcomes. At the same time, pain, walking capacity, knee ROM, and knee stability improved significantly in the WG. It is essential to balance the benefits of early weight-bearing and the risks of mechanical complications. Immediate weight-bearing after fracture fixation offers several gains, including superior healing, increased strength, and overall health and socioeconomic benefits [14]. However, reduction loss and post-traumatic osteoarthritis with early weight-bearing are often orthopaedic surgeons’ serious concerns [15]. As a result, patients undergoing ORIF for TP fractures are typically advised to restrict weight-bearing for several weeks [3]. There is limited scientific evidence, no explicit agreement on the ideal weight-bearing approach, or definitive protocols for post-operative rehabilitation following TP fracture plating. This study sought to answer this question as immediate postoperative full weight-bearing to tolerance did not negatively impact fracture fixation or articular collapse. These results are similar to previous retrospective research, which reported that patients who underwent early weight-bearing protocol had faster recovery and similar clinical outcomes and complications compared to patients who started weight-bearing after ten weeks of NWB [16]. Another study found no differences in the incidence of postoperative complications between the partial weight-bearing group and the NWB group in all Schatzker classification types [17]. The critical concern against early weight-bearing is the possibility of fracture displacement, so the case series of Schatzker type II fractures assessed the stability of the fracture, weight-bearing inducible fracture fragments displacements, and their migration over one year using radio-stereometric methods. The authors concluded that internal fixation with subchondral screws and buttress plates provided adequate stability to allow immediate post-operative partial weight-bearing without harmful consequences [18]. Their results align with previous reports using a locking screw plate that might also allow full or at least partial weight-bearing [19].

Previous research retrospectively analyzed data from 90 patients for Schatzker type I, II, IV & VI fracture patterns, and only one patient from the weight-bearing group had 4 mm joint depression, which did not progress after the first three month follow-up [20] In another retrospective study, 17 Patients were advised to load their limbs to a maximum of 20 kg during the first six weeks. A ratio of limb loading (affected to unaffected) was calculated at two, six and 12 weeks postoperative. Despite poor adherence to postoperative weight-bearing restrictions, no fractures demonstrated any measurable postoperative migration, and significant improvements were seen in all reported outcome measures over the first year. Significant improvements were observed in all patient-reported outcome measures over the first year [21]. Restricting weight-bearing status significantly increases energy expenditure, according to a study that investigated healthy volunteers and measured the Physiological cost index for each weight-bearing status. This should be considered when recommending such restrictions in clinical practice and encouraging early mobility [22]. These results contrast with previous recommendations and protocols where 39% of studies recommended four to six weeks of NWB, with a further for to six weeks of partial weight-bearing [23]. Another study extracted the data of 91 geriatric patients who suffered TP fracture retrospectively with a follow-up period of up to six years. The authors found that loss of condylar widening corrections was only significantly more remarkable for the early weight-bearing group compared to the NWB group [24].

Very few studies investigated the longer-term outcomes, with more favorable results for immediate weight-bearing with no difference in reoperations, articular incongruity, or development of osteoarthritis signs between the two groups in a minimum two-year follow-up. An example is the study that allowed partial to full weight-bearing in the circular fixator group immediately postoperatively [25]. Our study, the first prospective clinical trial comparing radiological outcomes of immediate weight-bearing versus NWB, adds evidence supporting full weight-bearing (to tolerance) in patients with surgically treated TP Schatzker types I-IV fractures by locking plates.

Our study’s limitations include a small subgroup sample size, single-center design, relatively short follow-up period, and the lack of monitoring of patient weight-bearing compliance. Future studies, including all Schatzker types, are needed with longer follow-ups.

## Conclusion

Immediate weight-bearing, as tolerated after open reduction and internal fixation of TP fractures (Schatzker I-IV) by locking plates, resulted in better clinical outcomes with no significant differences in the radiological measures or complication rates.

## Electronic supplementary material

Below is the link to the electronic supplementary material.

Supplementary Material 1

## Data Availability

No datasets were generated or analysed during the current study.
